# Impact of Cognitive Impairments on Health-Related Quality of Life in Schizophrenia

**DOI:** 10.3390/brainsci13020215

**Published:** 2023-01-28

**Authors:** Gurpreet Rekhi, Young Ern Saw, Keane Lim, Richard S. E. Keefe, Jimmy Lee

**Affiliations:** 1 Research Division, Institute of Mental Health, Singapore 539747, Singapore; 2 Department of Psychiatry and Behavioral Sciences, Duke University Medical Center, Durham, NC 27710, USA; 3 North Region & Department of Psychosis, Institute of Mental Health, Singapore 539747, Singapore; 4 Lee Kong Chian School of Medicine, Nanyang Technological University, Singapore 308232, Singapore

**Keywords:** cognitive impairments, health-related quality of life, quality of life, schizophrenia, cognitive factor

## Abstract

The impact of cognitive impairments on the health-related quality of life (HRQoL) in individuals with schizophrenia is unclear. The aim of this study was to examine the association between cognitive impairments and HRQoL in individuals with schizophrenia. A total of 609 individuals with schizophrenia were assessed on the Positive and Negative Syndrome Scale (PANSS) and a neurocognitive battery which comprised of the Wechsler Abbreviated Scale of Intelligence matrix reasoning, the Benton Judgment of Line Orientation Test, Continuous Performance Tests-Identical Pairs, and the Brief Assessment of Cognition in Schizophrenia. A cognitive factor *g* was derived from the neurocognitive battery. EuroQol five-dimensional (EQ-5D-5L) utility scores were derived from PANSS scores via a previously validated algorithm and used as a measure of HRQoL. Hierarchical multiple regression was conducted to examine the association between cognitive factor *g* and the EQ-5D-5L. Cognitive factor *g* (β = 0.189, t = 4.956, *p* < 0.001) was found to be significantly associated with EQ-5D-5L scores. Age (β = −0.258, t = −6.776, *p* < 0.001), sex (β = 0.081, t = 2.117, *p* = 0.035), and being employed (β = 0.091, t = 2.317, *p* = 0.021) were also significant predictors of EQ-5D-5L. Our results add to the extant literature on the burden cognitive impairments exact in individuals with schizophrenia. More research is needed to develop effective interventions for cognitive impairments in schizophrenia.

## 1. Introduction

Cognitive impairments are a core feature of schizophrenia. Composite cognitive scores in individuals with schizophrenia were reported to be 1.5 to 2.5 SDs below that of healthy controls [1,2]. Impairments in the cognitive domains of executive function, working memory, verbal memory, language, attention and speed, and vigilance were reported in individuals with schizophrenia [3,4,5]. Cognitive impairments in schizophrenia were consistently reported to be associated with real-life functioning, employment, and independence of living [6,7,8]. The negative impact of cognitive impairments on individuals with schizophrenia, coupled with the lack of available treatments, makes it an important unmet treatment need in schizophrenia [9].

In recent years, discussion around treatment outcomes in schizophrenia has widened beyond symptom amelioration to include personal recovery and focus on improving the Quality of Life (QoL) of individuals with schizophrenia [10]. QoL was suggested to be a multidimensional concept and was broadly defined as a person’s overall sense of wellbeing, functional status, satisfaction with his/her life circumstances, and access to resources and opportunities [11,12]. Improvement in a person’s QoL is a vital factor when assessing the effectiveness of any treatment intervention. Health-Related QoL (HRQoL), was proposed to encompass the effects of illness (e.g., schizophrenia) on QoL, i.e., how the individual perceives the effects of physical and mental health and illness on his/her functioning and psychological state, and the disability caused by the illness [11]. HRQoL measures were employed in the computation of quality-adjusted life-year (QALY) for comparisons across different psychiatric and non-psychiatric conditions and economic analyses [13].

Most existing studies on the burden of cognitive impairments in schizophrenia use QoL rather than HRQoL measures. Cognitive impairments were reported to be associated with a reduction in QoL [14,15,16,17,18]; a similar association was found when HRQoL measures were used [19,20]. On the other hand, some studies reported a very weak or no significant association between cognitive impairments and QoL in schizophrenia [21]. Further, some of the above studies found that only certain domains of cognition were associated with QoL [14,15,18] or HRQoL [19,20]. Additionally, a meta-analysis found small-to-moderate correlations between measures of cognition and objective QoL, but non-significant or inverse relationships between cognitive functioning and subjective QoL [22]. It was suggested that individuals with schizophrenia who showed higher cognitive functioning were more aware of their current predicaments or had sufficient cognitive awareness to compare their lives and situation against others. Consequently, they were more likely to be depressed and less satisfied [22]. Overall, there remain inconsistencies about the impact of cognitive functioning on the QoL in individuals with schizophrenia. Many of these studies used a small sample size (*n* < 100) which may affect the power of the study analyses [14,15,17,18,19,20,21]. Secondly, only correlational analysis was used to study the association between QoL or HRQoL and cognitive impairments in some studies [14,19] and the effect of other variables which could be associated with QoL was not controlled for.

Due to all the above stated factors, more research is needed on the burden of cognitive impairments in schizophrenia using larger sample sizes, multivariate analyses, and HRQoL measures. We have attempted to do so in the current study. This study aims to examine the association between cognitive impairments and HRQoL in individuals with schizophrenia. We hypothesized that cognitive impairments would be significantly and negatively associated with HRQoL in individuals with schizophrenia.

## 2. Materials and Methods

### 2.1. Setting and Study Participants

Participants consisted of both outpatients and inpatients recruited as part of the Singapore Translational and Clinical Research Psychosis (STCRP) program at the Institute of Mental Health, Singapore. Details of this cohort were reported previously [23,24]. The study was conducted from 2008 to 2011. Participants were included if they were of Chinese ethnicity, 21 to 55 years of age, completed a minimum of six years of primary school education, and had a diagnosis of schizophrenia ascertained on the Structured Clinical Interview for DSM-IV-TR Axis I Disorders [25]. Participants were excluded if they had a significant history of substance abuse, clinically significant neurological disease or injury, or were color blind. A total of 975 individuals diagnosed with schizophrenia were recruited. The PANSS data was incomplete or missing for 54 participants and data on one or more cognitive tasks was missing for 340 participants. Overall, 366 participants had missing data on either the PANSS or any of the cognitive measures, or both. Hence, only 609 participants were included in the current study. All the participants provided written informed consent before the study assessments. Ethics approval for the study was given by the National Healthcare Group’s Domain Specific Review Board.

### 2.2. Assessments

Demographic information was collected from the study participants. Participants completed a neurocognitive battery that included the Wechsler Abbreviated Scale of Intelligence (WASI) matrix reasoning [26], the Benton Judgment of Line Orientation Test [27], Continuous Performance Tests-Identical Pairs (CPT-IP) [28], and the Brief Assessment of Cognition in Schizophrenia (BACS) [29,30]. The BACS is a brief neurocognitive assessment designed to measure a participant’s speed of processing, problem-solving and reasoning, verbal memory, and working memory.

**Figure A1:**
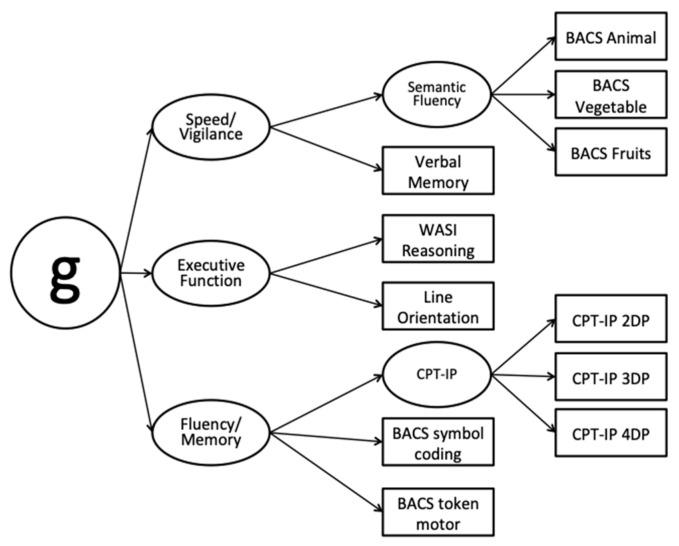
Cognitive Architecture of Individuals with Schizophrenia.

A factor analytic derived *g* was obtained and confirmed by our group previously [23] (See Appendix A Figure A1). To derive *g*, three latent variables were computed—speed/vigilance, executive function, and fluency/Memory. Neurocognitive scores were standardized using means and standard deviations from healthy controls. Speed/vigilance consisted of scores from the BACS semantic fluency and the BACS verbal memory task. Executive function was based on the WASI matrix reasoning and the Benton Judgement of Line Orientation Test. Finally, fluency/memory was calculated via the BACS symbol coding, BACS token motor, and the CPT-IP 2,3,4 d’ [23]. The factor-derived *g* score for each participant was obtained as an overall measure of cognition.

The PANSS is a 30-item semi-structured interview frequently used to determine the severity of symptoms in individuals with schizophrenia [31]. It has three subscales—positive, negative, and general psychopathology. The positive and negative subscales have seven items each, whereas the general psychopathology scale has sixteen items. Each item is rated on a Likert scale from 1 (absent) to 7 (extreme).

The EuroQol five-dimensional (EQ-5D-5L) assessment was used to assess HRQoL [32,33]. In clinical settings, clinicians might be unable to use specific HRQoL measures to assess the HRQoL of their clients due to paucity of time. Keeping this in mind, the use of mapping algorithms to convert commonly used clinical rating scales to generate utility scores and QALY for cost-utility analyses was suggested in previous literature [34,35]. A mapping algorithm for converting PANSS scores into EQ-5D-5L utility scores in schizophrenia was reported previously from a local sample [36] and employed in the current study. The algorithm is as follows: EQ-5D-5L utility = 1.3103 − 0.0044 × PANSS positive + 0.0025 × PANSS negative − 0.0146 × PANSS general psychopathology − 0.0029 × age + 0.0149 × female.

### 2.3. Statistical Analyses

All statistical analyses were performed via IBM SPSS Statistics 25. Mean and standard deviations were computed for continuous variables; and frequencies and percentages for categorical variables. To determine if cognitive factor g accounted for a statistically significant amount of variance in EQ-5D-5L and the amount of the unique variance, hierarchical multiple regression was employed. EQ-5D-5L was the dependent variable in the model. In the 1st step of the regression model, the covariates of age, sex, and employment status were entered. In the 2nd step, the cognitive factor *g* was included as the independent variable. Statistical significance was established at *p* < 0.05.

## 3. Results

### 3.1. Socio-Demographic and Clinical Characteristics of the Sample

The demographics and clinical characteristics of study participants are shown in Table 1. Around half of the participants were males (*n* = 323, 53.00%). Most of the participants were educated until secondary level (i.e., Grade 10) or above (*n* = 456, 74.88%). About half of the participants were employed (*n* = 291, 47.8%). The standardized scores on the neurocognitive battery for participants can be seen in Table 2. Standardized scores on speed/vigilance, fluency/memory and executive functioning were −1.96 (*SD* = 1.24), −2.16 (*SD* = 1.20), and −1.42 (*SD* = 1.26), respectively.

### 3.2. Association between Cognitive Factor g and PANSS-Derived EQ-5D-5L

The hierarchical multiple regression model was significant when the cognitive factor g was included, *F*(5,603) = 20.033, *p* < 0.001. The first step of the hierarchical multiple regression with only the covariates accounted for 10.2% of the variance, *F*(4,604) = 18.190, *p* < 0.001. In the second step, cognitive factor g accounted for a statistically significant 3.3% increase in variance, *F* Change(1,603) = 24.565, *p* < 0.001. In the second step of the hierarchical multiple regression model, older age (β = −0.258, *t* = −6.776, *p* < 0.001) was found to be significantly associated with lower EQ-5D-5L scores, whereas female sex (β = 0.081, *t* = 2.117, *p* = 0.035), being employed (β = 0.091, *t* = 2.317, *p* = 0.021), and a higher *g* (β = 0.189, *t* = 4.956, *p* < 0.001) were significantly associated with higher EQ-5D-5L scores. Overall, age and *g* had the largest size of effect (Table 3).

## 4. Discussion

This study aimed to examine the association between cognitive impairments and HRQoL in individuals with schizophrenia. The current study showed that individuals with schizophrenia with impaired cognitive functioning were more likely to have a lower HRQoL. The results supported our hypothesis that cognitive impairments were negatively associated with HRQoL in schizophrenia, and are consistent with previous research [19,20]. Theoretically, it was suggested that factors related to vulnerability to schizophrenia may affect both cognition and quality of life; therefore, the association between impairments in cognition and QOL in schizophrenia [20]. Further, the cognitive factor *g* in the current study only accounted for a small amount of variance in the EQ-5D-5L scores, which is in line with previous research [19]. A possible reason for this finding could be that the cognitive battery used in the current study did not include any test to assess social cognition, which was also suggested to be associated with quality of life in schizophrenia [37]. Nonetheless, as compared to other variables (except age) in the regression analysis, cognitive factor *g* had the largest size of effect.

A significant association was also found between age, sex, and employment status and the PANSS-derived EQ-5D-5L. We found that higher age was correlated with a lower EQ-5D-5L in schizophrenia, which aligns with previous research [38,39,40]. Older individuals with schizophrenia may be more dissatisfied with the prognoses of their illness versus their younger counterparts; this could explain the poorer QOL among the former [40]. Further, the findings in the current study were consistent with other studies that female sex was associated with higher QOL when compared to males with schizophrenia [41,42]. Previous evidence suggested that when compared to males, females with schizophrenia have a better response to medications, prognosis of illness, and social functioning [43]; this could explain why females have better QOL than males with schizophrenia. In addition, the current study found that individuals with schizophrenia who were employed were more likely to have a higher HRQoL when compared to unemployed individuals. Previous studies have noted a similar relationship between employment and QoL [39,44,45]. A workplace could provide a platform for individuals with schizophrenia to meet others and obtain social support, hence, leading to an increase in their QoL [46].

This study has some limitations. Firstly, as the PANSS-derived EQ-5D-5L was an aggregated score, it provided no information about impairments in the specific domains of HRQoL (e.g., mobility, usual activities, self-care, pain/discomfort, and anxiety/depression). Consequently, we were unable to determine if cognitive factor *g* might play a larger role in particular HRQoL domains. Secondly, the effect of symptoms, including depressive symptoms on HRQoL, could not be controlled for in the analyses because symptom scores were used to derive the EQ-5D-5L utility scores. Use of a separate measure to assess depressive symptoms could help to control for the effect of depressive symptoms on HRQoL. Thirdly, the sample used to derive the algorithm to obtain EQ-5D-5L scores from PANSS [36] consisted of not only Chinese but also Malay, Indian, and other ethnicities. Therefore, the use of this algorithm in the current study may induce some bias to HRQOL scores. Next, the effect of social cognition on HRQoL was not assessed in this study. Further, as this was a cross-sectional study, we could not examine the stability and the causality of the association between cognition and HRQoL in schizophrenia. Future studies with longitudinal design are needed to overcome these limitations. Next, most of the study participants were recruited from outpatient clinics and around half of the sample (53.9%) were either employed, homemakers or students. As such, the results of the current study might not be generalizable to those with severe illnesses. In addition, around one-third of the original study participants (*n* = 340) were excluded from this study analysis due to missing data on one or more of the neurocognitive tasks. Consequently, the study might be limited to individuals with more stable cognitive functioning as they were more likely to complete the neurocognitive battery.

This is one of the first few studies to investigate the viability of the PANSS-derived EQ-5D-5L. HRQoL measures are needed for economic evaluation of individuals with schizophrenia, but they are not commonly used in clinical practice. The current study suggested that in the absence of specific HRQOL measures, the PANSS-derived EQ-5D-5L might be a viable approach to examine HRQoL in individuals with schizophrenia. Only age, gender, and PANSS scores were used in the algorithm to derive HRQOL scores [36] and PANSS is commonly used to assess symptoms in clinical practice as well as clinical trials for schizophrenia. Nonetheless, one should still be aware of the potential limitations when the PANSS is converted to the EQ-5D-5L, as noted in limitations of the current study. Future studies could investigate the use of the original EQ-5D-5L and other HRQoL measures and compare their performance with the PANSS-derived EQ-5D-5L in schizophrenia.

## 5. Conclusions

In conclusion, the current study shows that cognitive impairments were associated with a lower HRQoL in individuals with schizophrenia. Cognitive impairments exact a significant burden on individuals with schizophrenia and remain an important unmet clinical need. We hope this study adds to the extant literature and spurs further research for interventions to improve cognitive function in schizophrenia.

## Figures and Tables

**Table 1:** Socio-demographic and clinical characteristics of the Participants (*n* = 609).

	*M* (*SD*) or *n* (%)
Age, years	38.98 (9.61)
Sex	
Male	323 (53.00)
Female	286 (47.00)
Duration of illness, years	15.23 (9.98)
Total Years of Education, years	12.01 (3.06)
Employment status	
Employed	291 (47.80)
Homemakers/Students	37 (6.10)
Unemployed	281 (46.1)
PANSS scores	
Positive	12.26 (5.25)
Negative	12.51 (5.37)
General Psychopathology	24.63 (7.00)
Total	49.39 (14.05)
EQ-5D-5L Utility Score	0.82 (0.12)
Cognitive Factor *g*	−2.02 (1.25)

PANSS, Positive and negative syndrome scale; EQ-5D-5L, EuroQol five-dimensional.

**Table 2:** Participants’ standardized scores on neurocognitive battery.

	*M*	*SD*
BACS Verbal Memory	−1.22	1.67
BACS Token Motor Task	−1.54	1.37
BACS Semantic Fluency Total	−1.20	1.05
BACS Semantic Fluency Animals	−0.85	0.93
BACS Semantic Fluency Vegetables	−0.85	0.93
BACS Semantic Fluency Fruits	−1.10	1.04
BACS Symbol Coding	−1.93	1.25
Benton Judgement of Line Orientation	−0.69	1.40
WASI Matrix Reasoning	−1.24	1.53
CPT-IP 2D’	−1.31	1.62
CPT-IP 3D’	−1.16	1.37
CPT-IP 4D’	−0.83	1.02

BACS, Brief Assessment of Cognition in Schizophrenia; WASI, Wechsler Abbreviated Scale of Intelligence; CPT-IP = Continuous Performance Test—Identical Pair.

**Table 3:** Summary of hierarchical multiple regression results.

Variables	*B*	*SE*	β	*t*	*p*	95.0% CI of B		Adjusted *R*^2^
						Lower	Upper	
*Step 1*								
Age	−0.003	0.000	−0.271	−7.012	<0.001	−0.004	−0.003	10.2
Sex	0.018	0.009	0.078	1.998	0.046	0.000	0.018	
Employment status								
Homemaker/Student	0.043	0.020	0.089	2.203	0.028	0.005	0.081	
Employed	0.027	0.009	0.117	2.937	0.003	0.009	0.045	
*Step 2*								
Age	−0.003	0.000	−0.258	−6.776	<0.001	−0.004	−0.002	13.5
Sex	0.019	0.009	0.081	2.117	0.035	0.001	0.036	
Employment status								
Homemaker/Student	0.037	0.019	0.077	1.926	0.055	−0.001	0.075	
Employed	0.021	0.009	0.091	2.317	0.021	0.003	0.039	
Cognitive Factor *g*	0.017	0.004	0.189	4.956	<0.001	0.011	0.024	

Step 2: *F* Change(1,603) = 24.565, *p* < 0.001.

## Data Availability

Data cannot be shared publicly because we did not have consent from participants to do so and this was not in our study IRB proposal. Data from this study consists of sensitive information such as presence of mental illness and socioeconomic status. Further, the local regulatory authorities require both an ethics approval and a data sharing agreement with the other party to be in place before de-identified data can be shared. This is a legal requirement. Readers who have any questions pertaining to the study can write to: imhresearch@imh.com.sg.
